# Assessing left ventricular systolic function with ejection fraction: using a double-edged knife as a hammer

**DOI:** 10.1186/s13613-019-0582-z

**Published:** 2019-10-02

**Authors:** M. Ignacio Monge García, Maurizio Cecconi, Michael R. Pinsky

**Affiliations:** 1 Unidad de Cuidados Intensivos, Hospital Universitario SAS de Jerez, C/Circunvalación, s/n, 11407 Jerez de la Frontera, Spain; 2 Department Anaesthesia and Intensive Care Units, Humanitas Research Hospital, Humanitas University, Milan, Italy; 3 Department of Critical Care Medicine, University of Pittsburgh School of Medicine, Pittsburgh, USA

Dear Editor,

We have read with interest the paper about the potential limitations of arterial d*P*/d*t*_max_ for assessing left ventricular (LV) contractility [1]. In this study, the authors aimed to evaluate the performance of femoral d*P*/d*t*_max_ from tracking changes in LV systolic function during different therapeutic interventions. They have studied 19 patients with circulatory shock and concluded that femoral d*P*/d*t*_max_ was an unreliable estimate of LV systolic function as it was markedly affected by arterial load, particularly during changes in norepinephrine dose.

As the authors have mentioned our recent study about this specific topic [2], we would like to highlight some potential issues that we believe should be considered when interpreting their results.

First and most important, the use of LV ejection fraction (LVEF) as a marker of LV systolic function. As the authors themselves declared, LVEF cannot be considered as a pure marker of LV systolic function, as it is also greatly influenced by the afterload conditions [3]. In this regard, we have recently published a detailed analysis of the effects of different loading and contractile conditions on LVEF [4]. In that study, we showed that LVEF poorly tracked LV contractility changes when using LV end-systolic elastance (Ees) as a load-independent measure of cardiac contractility [5], especially when afterload was altered.

To demonstrate the potential impact of this phenomenon on the current study, we have performed a concordance analysis using data from our previous study (Fig. 1) [2]. These four-quadrant plots represent the agreement in the directional change of the variations of two variables. The upper plot represents the agreement in LVEF and LV contractility, as assessed by Ees. The graph in middle shows the agreement between femoral d*P*/d*t*_max_ and Ees. And the last one, the concordance between femoral d*P*/d*t*_max_ and LVEF, just as the authors have performed in the current study. It is clear from this analysis that LVEF was a poor index of LV contractility during changes in afterload conditions (upper plot), as LVEF changes in the opposite direction to variations in LV contractility. However, femoral d*P*/d*t*_max_ was able to track contractility changes during afterload interventions (middle plot), even if changes in arterial load conditions could potentially affect femoral d*P*/d*t*_max_. Finally, comparing femoral d*P*/d*t*_max_ against LVEF, i.e., reproducing the author’s hypothesis and methodology, only proved that LVEF was unable to track changes in LV contractility when afterload was primarily affected. So, the use of LVEF as a surrogate for defining LV systolic function in the current study could explain the discrepancies with our previous results, which were based on a load-independent measure of contractility such as the LV end-systolic elastance. Therefore, for a physiological study aimed at evaluating LV contractility, the use of LVEF cannot be recommended.

**Fig. 1:**
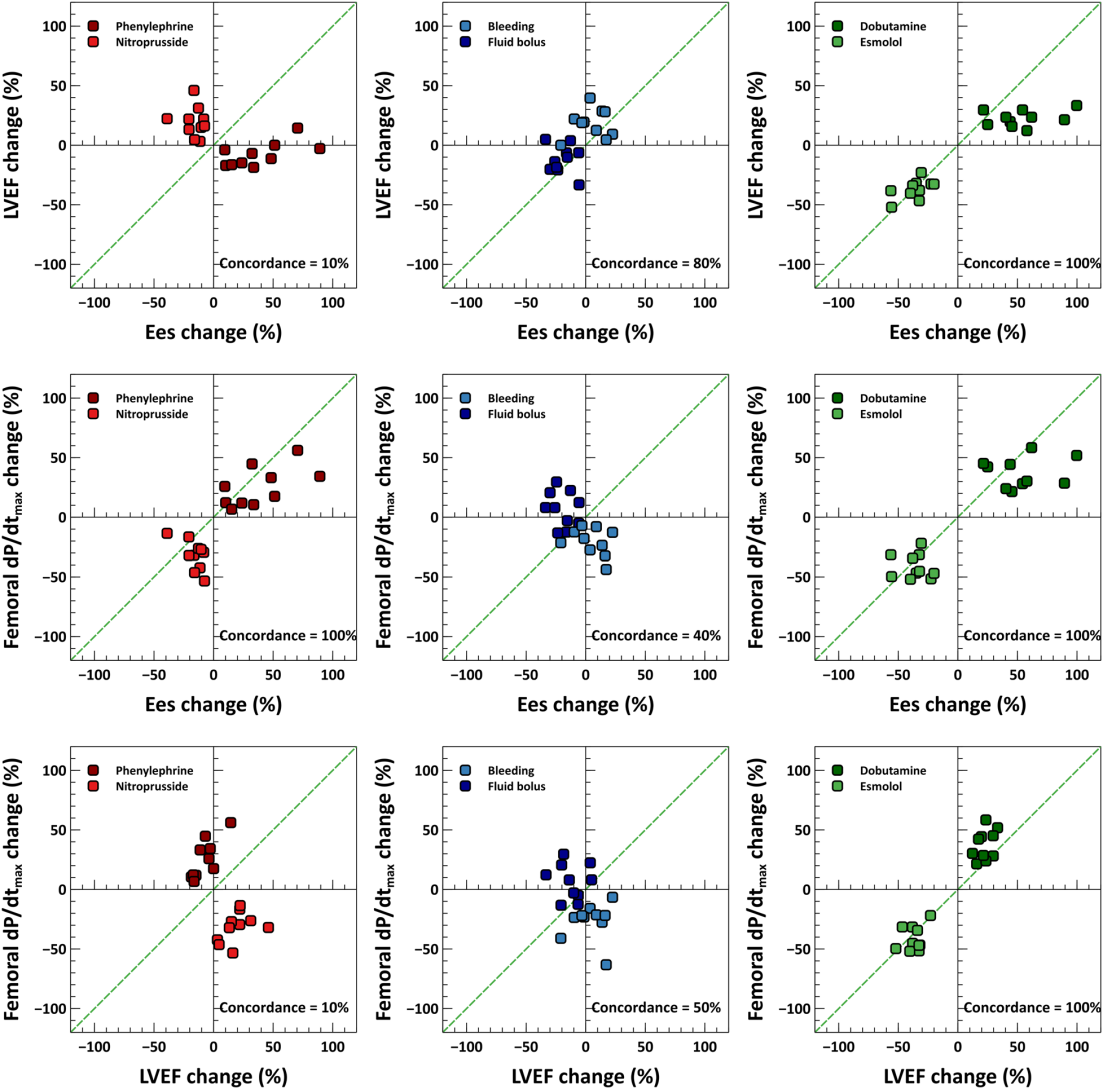
Concordance analysis. Four-quadrant plots showing the relationship between the percentage changes in left ventricular ejection fraction (LVEF) and left ventricular end-systolic elastance (Ees) (upper plot), femoral d*P*/d*t*_max_ and Ees (middle plot), and femoral d*P*/d*t*_max_ and LVEF (lower plot). Excellent trending capability is considered when ≥ 90% of the points lie in the right-upper and left-lower quadrants

So, unfortunately, although we acknowledge that evaluating LV contractility at the bedside is not an easy task, we believe that the current study could be weakened by this methodological assumption. As our previous study demonstrated, femoral d*P*/d*t*_max_ was able to track contractility changes even if the intervention was primarily to alter arterial tone or contractility varied. Since femoral d*P*/d*t*_max_ is readily available from the femoral arterial waveform, this information is clinically valuable.

## Data Availability

The data that support the findings of this study are available from the corresponding author upon reasonable request.
